# Interspecific sensitivity of bees towards dimethoate and implications for environmental risk assessment

**DOI:** 10.1038/srep34439

**Published:** 2016-09-30

**Authors:** Philipp Uhl, Lea A. Franke, Christina Rehberg, Claudia Wollmann, Peter Stahlschmidt, Lukas Jeker, Carsten A. Brühl

**Affiliations:** 1 University of Koblenz-Landau, Institute for Environmental Sciences, Fortstrasse 7, 76829 Landau, Germany; 2 Dr. Knoell Consult Schweiz GmbH, Riehenstrasse 43, 4058 Basel, Switzerland

## Abstract

Wild and domesticated bee species are exposed to a variety of pesticides which may drive pollinator decline. Due to wild bee sensitivity data shortage, it is unclear if the honey bee Apis mellifera is a suitable surrogate species in the current EU risk assessment scheme. Furthermore, the underlying causes for sensitivity differences in bees are not established. We assessed the acute toxicity (median lethal dose, LD50) of dimethoate towards multiple bee species, generated a species sensitivity distribution and derived a hazardous dose (HD5). Furthermore, we performed a regression analysis with body weight and dimethoate toxicity. HD5 lower 95% confidence limit was equal to honey bee mean LD50 when applying a safety factor of 10. Body weight proved to be a predictor of interspecific bee sensitivity but did not explain the pattern completely. Using acute toxicity values from honey bees and a safety factor of 10 seems to cover the interspecific sensitivity range of bees in the case of dimethoate. Acute endpoints of proposed additional test species, the buff-tailed bumblebee Bombus terrestris and the red mason bee Osmia bicornis, do not improve the risk assessment for the entire group. However, this might not apply to other insecticides such as neonicotinoids.

Agricultural crops and wild plants are mostly pollinated by insects and bees play a major role. Wild and domesticated bee species are affected by multiple environmental factors^1^. Since the last century the USA and Europe have experienced substantial losses of domesticated honey bee (*Apis mellifera*) colonies and simultaneous decline in wild bee diversity^1^,^2^,^3^,^4^,^5^. In Germany 52% of wild bee species are included in the Red List^6^.

Decline of pollinator species might be related to pesticide use in agricultural landscapes amongst other factors such as parasites and habitat loss^1^. Honey bees have received some attention in terms of their sensitivity towards pesticides^7^,^8^ and are included in the regulatory risk assessment framework of the placement of pesticides on the market (Regulation (EC) 1107/2009). It was recently suggested that toxicity towards wild bees could be extrapolated from honey bee data. In a meta-analysis, Arena *et al*.^9^ found that in most cases wild bee species are less sensitive to common insecticides than honey bees when comparing LD50 values obtained from acute toxicity studies. This was consistent for five out of six tested insecticide classes, whereas wild bees displayed equal to higher sensitivity to neonicotinoids (median factor 1.06). Since relative susceptibility patterns vary for different insecticides, it is difficult to extrapolate acute toxicity data of a specific insecticide from the honey bee to a specific wild bee species using the current data^10^,^11^. Moreover, recent field studies on oilseed rape revealed that deducing responses from honey bee populations to wild bees may not be adequate in realistic exposure scenarios either^12^,^13^. Interspecific susceptibility patterns towards insecticides seem to be substance-specific at least at generic level^10^,^14^. Indicators for different sensitivities of bee species towards insecticides are not clearly established. Body weight and size are often stated to be predictive traits but there are other possible factors such as metabolism and cuticular physiology. Since only few wild bee species have been subject to ecotoxicological studies, reliable evidence of the relationship between sensitivity and such traits remains to be provided^9^.

Currently, the honey bee is the only pollinator species that is required to be evaluated in the EU pesticide risk assessment scheme^15^. However, wild bee species such as bumble bees and solitary bees differ substantially from the honey bee in their ecological properties, e.g. sociality, life cycle, behaviour, which might affect their population responses. Pesticide effects on solitary bee populations and to an extent even bumble bee colonies might be more pronounced than on honey bees since effects on individuals cannot be buffered by sheer numbers as in the hive of a superorganism^9^,^13^. Participants of a Society of Environmental Toxicology and Chemistry (SETAC) 2011 workshop in Pensacola (USA) pleaded for evaluating pesticide effects (lethal and sublethal) towards non-*Apis* species in laboratory, semi-field and field studies^16^. The European Food Safety Authority (EFSA) also identified a lack of information on the sensitivity of bumble bees and solitary bees^17^. They proposed to include the buff-tailed bumblebee *Bombus terrestris* and the red mason bee *Osmia bicornis* into EU pesticide risk assessment. In the current lower tier testing scheme, pesticides are categorised as having a low risk towards bees through contact exposure when the quotient of application rate and contact LD50 of the surrogate species, the honey bee is lower than 50^18^. EFSA^17^ proposed an additional assessment factor of 10 to account for interspecific differences in bee sensitivity. They referred to Arena *et al*.^9^ who found a factor of 10 to be protective in 95% of all cases in a meta-analysis of multiple insecticides, comparing endpoints of the honey bee and 19 wild bee species, 9 of which are tropical.

The species sensitivity distribution (SSD) is one approach to infer from laboratory test results on the effects that a pesticide has on bee species communities in the agricultural landscape. The underlying idea of the SSD is that interspecific sensitivity follows a statistical distribution. By fitting a suitable distribution to the data the dose at which 5% of species in a community are affected by a pesticide (HD5) can be derived^19^. To ensure a proper level of safety, i.e. reduce uncertainty, it was recommended to use the lower 95% confidence limit of the HD5 (lower limit HD5)^20^,^21^. To establish a SSD ecologically representative and comparable toxicity data are needed, as well as an appropriate statistical analysis method^21^,^22^.

In order to adequately assess the risk pesticides pose to bees a comprehensive database is needed. Sensitivity data for European bee species are scarce, covering only a few species that are bred for pollination services so far. The aim of the present study was to measure sensitivity of multiple bee species towards one insecticide to study interspecific sensitivity variability in bee species. We chose species that occur in the European agricultural landscape. These species may forage on crops and are therefore potentially exposed to insecticides in the field. We chose dimethoate as it is used as toxic reference in honey bee acute toxicity studies. Our first goal was to collect sufficient data from dose-response experiments to generate a SSD and deduce the effect of dimethoate on wild bee species. Subsequently, we compared the lower limit HD5 to the honey bee contact LD50 divided by 10 as proposed by EFSA. This enabled us to ascertain if the honey bee is a suitable surrogate organism for all bee species. Furthermore, we assessed if this safety factor covers the sensitivity range of wild bee species. Secondly, the sensitivity and weight data of multiple bee species was evaluated to deduce if body weight is a predictor of bee sensitivity.

## Results

### Species sensitivity distribution

We studied the effect of dimethoate on 5 European bee species that are abundant in the agricultural landscape. All species are categorised under “least concern” in the Red List^6^. Dimethoate sensitivity varied substantially between bee species in the following decreasing order (note that some species occur twice since there is no statistically significant difference of their LD50 to values of two other species that are different): *L. malachurum* = *A. flavipes* > *A. flavipes* = *C. hederae* = *O. bicornis* ♂ > *O. bicornis* ♀ = *B. terrestris* (Table 1, Supplementary Table S3, Fig. S4). However, when examining LD50 values at per fresh weight basis the order changes to: *C. hederae* = *A. flavipes* = *L. malachurum* > *A. flavipes* = *L. malachurum* = *B. terrestris* > *O. bicornis* ♂ = *O. bicornis* ♀ (Table 1, Supplementary Table S4, Fig. S5). Calculated per dry weight, sensitivity order changes again: *C. hederae* = *A. flavipes* > *A. flavipes* = *L. malachurum* > *B. terrestris* = *O. bicornis* ♂ > *O. bicornis* ♀ (Table 1, Supplementary Table S5, Fig. S6). *O. bicornis* ♀ was always among the most resistant species whereas *A. flavipes* was always among the most sensitive. *O. bicornis* ♀ were less sensitive than *O. bicornis* ♂ (Supplementary Table S3).

**Figure 1:**
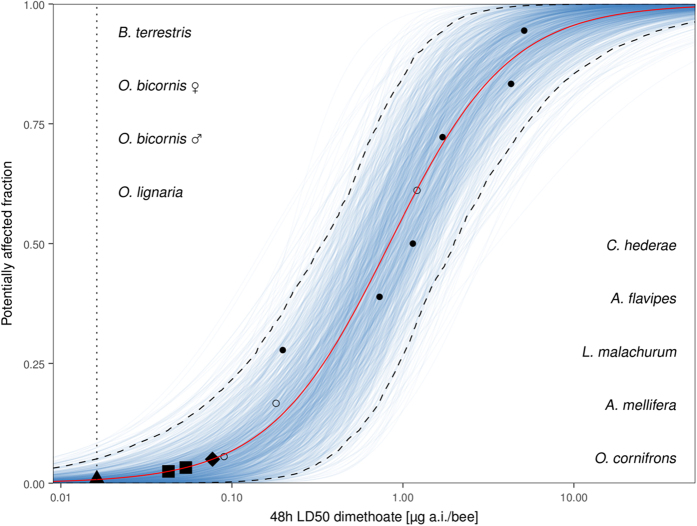
Species sensitivity distribution of dimethoate calculated from multiple bee species’ sensitivity (red line). ● & ○ denote 48 h LD50 values of bee species (○ are literature values). Species names are aligned by sensitivity in ascending order from bottom to top on the same y-axis coordinate as their respective ●/○. Dashed lines enclose parametric bootstrap 95% CI (1000 iterations). Blue, transparent lines display all parametric bootstrap samples. ■ marks the HD5 value, ▲ the lower limit HD5 and ■ the extrapolated LD50 values of *Hylaeus gredleri* ♂ and *Nomioides minutissimus* ♀. The proposed regulatory threshold of honey bee LD50/10 is indicated by the dotted line. LD50 values for *A. mellifera*, *O. cornifrons* and *O. lignaria* were taken from other studies (Supplementary Table S7).

HD5 was calculated to be 0.08 μg a.i./bee and the lower limit HD5 0.02 μg a.i./bee (Fig. 1, Supplementary Table S6). The lower limit HD5 is equal to the mean 48 h LD50 for *A. mellifera* calculated from literature data (0.18; Supplementary Table S7) divided by a safety factor of 10.

### Weight-sensitivity regression

**Figure 2:**
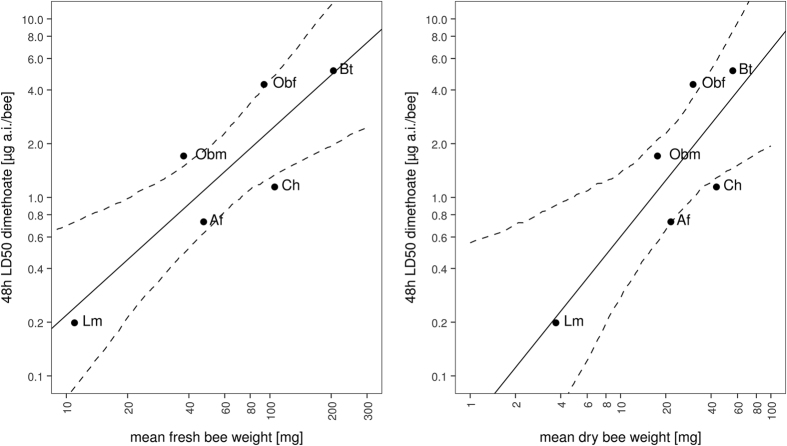
Relationship between bee weight (fresh and dry) and sensitivity towards dimethoate. Dots mark weight and sensitivity of the following species: Lm - *Lasioglossum malachurum*, Af - *Andrena flavipes*, Ch - *Colletes hederae*, Obm - *Osmia bicornis* ♂, Obf - *Osmia bicornis* ♀, Bt - *Bombus terrestris*. Both axes on logarithmic scale. Dashed lines enclose parametric bootstrap 95% CI (1000 iterations).

The studied bee species cover a wide weight range (Supplementary Table S2, Figs S2, S3). Workers from the heaviest species, *B. terrestris* (205 mg), were on average 19 times heavier than females from the lightest species, *L. malachurum* (11 mg; Wilcoxon rank sum test, p < 0.001). Body weight did influence wild bee species’ dimethoate sensitivity. We found a linear relationship of 48 h LD50 and weight (fresh and dry) when analysing the collected wild bee toxicity data (Fig. 2). This relationship is best described by a power function (exponential function of the general form 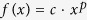; Table 2). However, incorporating literature values of *A. mellifera*, *O. lignaria* and *O. cornifrons* (Supplementary Table S7) into the model resulted in considerable decline in model fit. We extrapolated the 48 h LD50 values of two small German bee species (*Hylaeus gredleri* and *Nomioides minutissimus* ♀) to be 0.05 and 0.04 μg a.i./bee, respectively. These LD50 values are situated between the HD5 and the lower limit HD5 (Table 1, Fig. 1).

## Discussion

Suitability of *A. mellifera* as the sole surrogate species in acute toxicity testing was questioned by EFSA^17^. To reduce uncertainty additional bee species could be incorporated in pesticide risk assessment. The OECD honey bee guideline for acute contact toxicity testing requires the use of young adult worker bees of similar age^23^. It is not exactly stated how old bees should be which may lead to variation in age across research facilities. Since cuticular resistance and detoxification capacity develop with age but not before eclosion in honey bees^24^,^25^,^26^ different susceptibilities might be obtained from honey bee tests. Young solitary bees may even be relatively less susceptible due to a fully matured cuticle and already elevated antioxidant enzyme levels before eclosion^27^,^28^. Consequently, the honey bee may be a sufficient surrogate organism in some cases at least in lower tier testing with contact exposure. In any case bee age should be exactly defined in lower tier testing guidelines to reduce variability of generated LD50 values.

For reasons of reproducibility and costs of laboratory studies the SSD approach can be an acceptable compromise to higher tier testing. It produces ecologically relevant results which might be used as additional data, or an alternative to complex and cost-intensive semi-field or field studies^20^. However, the significance of SSD results for more complex systems has only been studied in aquatic experiments. There is a need to verify if this holds true for terrestrial settings. One conceptual shortcoming of the HD5 as a toxic endpoint is that it deems the most sensitive species expendable. However, those species might share the same ecological niche. In our case sensitive species are likely to be small species when considering the weight-sensitivity relationship (Fig. 2). When extrapolating toxicity of two of the smallest bee species in Germany with our weight-sensitivity regression model LD50 values were still higher than the lower limit HD5. Therefore, we cannot confirm that small, sensitive bee species are put at risk by using the HD5 in risk assessment.

In our study the safety factor of 10 recommended by EFSA^17^ seems to cover the acute sensitivity range of wild bee species. We modeled dimethoate sensitivity of multiple bee species and found that the lower limit HD5 is equal to the mean 48 h LD50 value of honey bees divided by this safety factor (Fig. 1). Therefore, testing the honey bee and employing a safety factor of 10 seems to be adequate for lower tier risk assessment of dimethoate. However, bee species acute toxicity data we inferred from are still limited. Dimethoate is a well-studied insecticide that the honey bee is rather sensitive to^9^. For neonicotinoids, however, Arena *et al*.^9^ reported several studies where other bee species were at least as susceptible as the honey bee. Therefore, a safety factor of 10 might not encompass interspecific sensitivity in the case of those insecticides. There still is reasonable doubt that the honey bee is a feasible surrogate for all bee species since relative sensitivities of bee species vary with each pesticide^9^,^11^. The additional testing of a bumble bee and a solitary species was proposed by EFSA^17^ to reduce uncertainty. We argue that test species should be chosen according to their sensitivity and ecological relevance. The two species (*B. terrestris*, *O. bicornis*) recommended by EFSA^17^ were the least sensitive towards the toxic reference dimethoate in our experiments (LD50s 28.5 and 23.8 times higher than honey bee). Moreover, *B. terrestris* was also generally less sensitive than the honey bee in the studies surveyed by Arena *et al*.^9^ and Sanchez-Bayo & Goka^29^. Both species are commercially bred for pollination services in agricultural systems where pesticides are frequently used (*O. bicornis* in e.g. apple orchards, *B. terrestris* in greenhouses). Therefore, they can be procured in high numbers for testing and can be handled quite well in the laboratory. However, it is unclear which additional information is to be gained from testing rather pesticide-resistant species. To substantially reduce uncertainty in lower tier risk assessment sensitive species should be studied. To achieve that goal a comprehensive database of interspecific sensitivity of bees is needed. Furthermore, differences in responses of bee species to pesticides should also be considered in higher tier testing. Pesticide impact on bee species in the field is governed by ecological differences as shown by Rundlöf *et al*.^13^. We propose that bee risk assessment should rather focus more on testing multiple species in realistic settings than in the laboratory.

Several traits are assumed to determine interspecific sensitivity differences in bees, mainly body size and weight. However, data on bee species sensitivity is scarce which hinders reliable inference on predictive factors^9^. We evaluated sensitivity and weight data of multiple bee species to deduce if body weight is a predictor of bee sensitivity. Comparing 48 h LD50 values of five European bee species we found that dimethoate toxicity increases with decreasing bee species weight (Table 2, Fig. 2). Incorporating literature values considerably decreased model fit. The reason might be laboratory-specific differences in bee health status, e.g. pathogen or virus levels, as well as varying sensitivity of bee strains from different parts of the world^30^. Furthermore, body weight and sensitivity data could only be procured from separate studies. Besides the traits summarized by Arena *et al*.^9^ there are additional factors that may substantially affect bee sensitivity towards pesticides. Amongst other things uptake, metabolism and excretion of topically applied pesticide solutions define their toxic impact. The generally accepted uptake mechanism is that pesticides are diluted in both layers of the cuticle and subsequently distributed in the hemolymph to reach the central nervous system^31^. Cuticular maturation may have an effect on pesticide uptake since permeability decreases during this hardening and darkening process. Cuticular hydrocarbon profiles differ between honey bee pupae, newly-emerged workers and adult foragers^24^. Unlike in honey bees, solitary bee cuticle is fully developed at eclosion^27^. Interspecific differences in cuticular composition may be an additional factor but there are no studies on that subject. Once a pesticide has entered the insect body, its actual toxic effect on the insect depends on the organism’s capacity to metabolize and subsequently excrete it. Such detoxification processes are controlled by enzyme activity. Common European bee species such as the *B. terrestris*, the solitary bee *Megachile rotundata* and the honey bee *A. mellifera* were reported to show similar levels of genes that are associated with detoxification processes^32^. Nevertheless, there are interspecific differences in the buildup of these enzyme levels during bee development. In adult honey bees the detoxification capacity is quite low at eclosion and increases as they become foragers^25^,^26^. In the solitary bee *O. bicornis*, however, antioxidant enzyme levels are already building up before eclosion^28^. Our data suggest that body weight is a governing factor of bee sensitivity towards dimethoate but it remains unclear if this holds true for all pesticides in general. Further research on interspecific sensitivity of bees is needed.

In this study we computed a SSD from dimethoate acute toxicity data of wild bee species. The derived lower limit HD5 was equivalent to the honey bee LD50 value divided by a safety factor of 10. This value also encompasses two of the smallest wild bee species which LD50 values were calculated from a weight-sensitivity relationship. For dimethoate no further information is gained by conducting acute laboratory tests with the two wild bee species *B. terrestris* and *O. bicornis* as suggested by EFSA. We recommend to investigate wild bee toxicity for other insecticide groups and reconsider the proposed acute testing scheme. Adding wild bee species to environmental risk assessment for pesticides seems to be important when considering field-relevant effects where differences in sociality and behaviour affect sensitivity, but not so when testing on an organism level in a laboratory.

## Methods

### Insecticide

We used a formulation of dimethoate (Perfekthion^®^, BASF, 40% a.i. (w/w)). It is an organophosphate insecticide which acts on the nervous system by inhibiting acetylcholinesterase and is highly toxic to honey bees^33^.

### Provision of test species

Five different bee species were used: the buff-tailed bumble bee (workers) *Bombus terrestris* (Linneaus), the red mason bee (♀ & ♂) *Osmia bicornis* (Linneaus), the sweat bee (♀) *Lasioglossum malachurum* (Kirby), the mining bee (♀) *Andrena flavipes* (Panzer) and the ivy bee (♀) *Colletes hederae* Schmidt & Westrich. Medium-sized *B. terrestris* colonies (60–80 workers) were obtained from a commercial breeder (Biofa AG, Rudolf-Diesel-Str. 2, 72525 Münsingen, Germany). *O. bicornis* were ordered as cocoons (WAB-Mauerbienenzucht, Sonnentauweg 47, 78467 Konstanz, Germany). Since males and females of *O. bicornis* were available, we also tested males of this species to infer on sex-specific sensitivity. All other species were caught at feeding grounds or nesting sites in the agricultural landscape around Landau, Germany with permission of regional authorities. Collected bees were examined to be viable and morphospecies were confirmed by visual inspection. All bee species were kept in an environmental chamber under experimental conditions, i.e. same environmental conditions, test cages, food etc., until the experiment was started. All species that were caught were collected on the day before test start so that the bees could acclimatise to experimental conditions. *O. bicornis* cocoons were put in the environmental chamber under test conditions for bees to eclose. It took around 3 days for enough males to emerge and around 5 for females. *B. terrestris* workers were collected from the colonies the day before test start. Further information on wild bee collection and identification can be found in the Supplementary Information.

### Experimental Procedure

Acute, contact toxicity tests were performed with all test species. All tests were conducted according to the ringtest protocol for solitary bee acute contact toxicity developed by the International Commission for Plant-Pollinator Relationships (ICPPR) with minor modifications in some tests that are noted below^34^. Before the experiment, bees were fed *ad libitum* with sucrose solution 50% (w/w) through plastic syringes. Bees were transferred to test cages (1 L plastic boxes sealed with a perforated lid) the day before application to acclimatize overnight. In the case of *B. terrestris* and *O. bicornis* 30 bees per treatment were set up (10 per cage, n = 3). The remaining species could not be collected in such large quantities in the agricultural landscape. Consequently, the number of bees per cage had to be reduced in these tests. Fifteen *L. malachurum* females per treatment were tested (5 per cage, n = 3). For *A. flavipes* and *C. hederae* the number of bees per treatment was 9 (3 per cage, n = 3). Environmental conditions were set to 8:16 h day/night rhythm (light intensity <10 lux at day), 60% humidity and 21 °C. Temperature for *B. terrestris* and *L. malachurum* was increased to 25 °C to better accommodate them following EFSA recommendations^17^. Bees were anaesthetised for the transfer to the test cages and for the application. All species were chilled at 4 °C and put in a petri dish on ice for the application, whereas bumble bees were anaesthetised with CO_2_ since chilling did not calm them down to allow safe handling. Moribund bees were rejected and replaced by healthy bees prior to the test start. Wet and dry weight were determined for all bee species: Anaesthetised *B. terrestris* and *O. bicornis* specimens were weighed before treatment application. Individuals of all other species were weighed after the experiment to avoid loss of bees due to excessive handling since the number of specimens was already limited. We tested six treatments per bee species: a control of deionised water containing 0.5% (v/v) wetting agent (Tween 80; Carl Roth GmbH + Co. KG) and five dimethoate treatments. Dimethoate doses of 1.25, 2.5, 5, 10 and 20 μg a.i./bee were chosen for *B. terrestris*. *O. bicornis* specimens were applied with 0.625, 1.25, 2.5, 5 and 10 μg a.i./bee. For individuals of the remaining species we used 0.0896, 0.224, 0.56, 1.4 and 3.5 μg a.i./bee. Dimethoate solutions were prepared by diluting the respective concentration in deionised water containing 0.5% wetting agent (Tween 80). Bees were applied with 1 μL or 5 μL in case of *B. terrestris* on the dorsal side of the thorax between the neck and wing base using a Hamilton micro syringe (Hamilton Bonaduz AG). A paper tissue was inserted into test cages after treatment solution was fully absorbed (10 to 15 min) to provide a hiding place. Bumble bees had to be anaesthetised once more for that procedure. Following the application bees were returned to the environmental chamber and fed 50% sucrose solution *ad libitum*. After 48 h mortality was assessed. For *O. bicornis* ♀ 3 separate test runs were performed. In all 8 experiments control mortality was ≤10% except for *B. terrestris* (13%) and *A. flavipes* (22%). A subsample of 28 bees of all species were dried afterwards at 60 °C for 48 h and weighed again. Furthermore, samples of treatment solutions were chemically analysed to verify actual treatment doses for all *B. terrestris* and *O. bicornis* ♀ experiments (see Supplementary Information).

### Statistical analysis

Median lethal dose values (LD50) were calculated for all tested species by fitting a dose-response model to the data. Models were chosen by visual data inspection and using Akaike information criterion (AIC). Control mortality was corrected for by using Abbott’s formula^35^. Where multiple LD50 values were available a geometric mean LD50 was computed. Interspecific differences in sensitivity were analysed by performing hypothesis tests using the confidence interval (CI) overlap method (Bonferroni-adjusted) described in Wheeler *et al*.^36^. A species sensitivity distribution (SSD) was fitted to 48 h LD50 values of all examined species^19^. From that distribution we derived the 5% hazardous dose (HD5) and calculated its parametric bootstrap 95% confidence intervals (CIs, 5000 iterations) to obtain the lower limit HD5. To check for a dependency of bee sensitivity and weight we fitted a linear model using 48 h LD50 values as response and fresh or dry weight as predictor variable. LD50 literature values of comparable studies for *A. mellifera*, *O. lignaria* and *O. cornifrons* were included in dose-response modelling and regression analysis (Supplementary Table S7). Furthermore, we calculated fresh and dry weight-normalised LD50 to facilitate comparability of our results with other studies. Dimethoate effects on two of the smallest German bee species (*Hylaeus gredleri* ♂, *Nomioides minutissimus* ♀, personal communication, Matthias Kitt, ecological consultant, Raiffeisenstraße 39, 76872 Minfeld, GERMANY) were estimated using the weight-sensitivity regression model. These were compared to the calculated HD50. Dry weights were obtained from pinned specimens. All statistical analyses were conducted with R 3.1.2^37^. We used the “drc” package^38^ for dose-response modelling and “fitdistrplus”^39^ for fitting the SSD.

## Additional Information

**How to cite this article**: Uhl, P. *et al*. Interspecific sensitivity of bees towards dimethoate and implications for environmental risk assessment. *Sci. Rep.* **6**, 34439; doi: 10.1038/srep34439 (2016).

## Supplementary Material

Supplementary Information

## Figures and Tables

**Table 1:** Dimethoate sensitivity of studied bee species.

Species	Mean fresh weight	Mean dry weight	LD50	95% CI	LD50	95% CI	LD50	95% CI
					Fresh weight-normalised		Dry weight-normalised	
	[mg]	[mg]	[μg a.i./bee]		[μg a.i./g bee]		[μg a.i./g bee]	
*Lasioglossum malachurum*	11.0	3.7	0.20	0.16–0.24	18.08	14.70–21.46	53.40	43.42–63.37
*Andrena flavipes*	47.3	21.6	0.73	0.07–1.39	15.44	1.57–29.31	33.78	3.44–64.11
*Colletes hederae*	105.5	43.4	1.14	0.72–1.57	10.84	6.83–14.85	26.35	16.61–36.09
*Osmia bicornis* ♂	37.7	17.6	1.71	1.37–2.04	45.27	36.31–54.22	96.90	77.73–116.07
*Osmia bicornis* ♀	93.6	30.4	4.29	3.72–4.91	45.89	39.80–52.47	141.46	122.68–161.73
*Bombus terrestris*	205.0	55.8	5.13	4.10–6.15	25.00	20.00–30.00	91.87	73.49–110.26
*Nomioides minutissimus* ♀*	NA	0.8	0.04	NA	NA	NA	NA	NA
*Hylaeus gredleri* ♂*	NA	1.0	0.05	NA	NA	NA	NA	NA

*Toxicity values are extrapolated using the computed weight-sensitivity relationship.

**Table 2:** Summary of different models to predict LD50 values from bee weight.

Model	Predictor	Literature values	R^2^	Parameter	Estimate	SE	p
log_10_(*y*) = *a* · log_10_(*x*) + *b*	fresh weight	yes	0.34	a	0.8087	0.4623	0.131
				b	−1.4550	0.8579	0.141
		no	0.76	a	1.0339	0.2879	0.022
				b	−1.6938	0.5216	0.031
	dry weight	yes	0.37	a	1.1068	0.5512	0.085
				b	−1.5846	0.7591	0.075
		no	0.70	a	1.0490	0.3399	0.037
				b	−1.2693	0.4723	0.055

Models vary in predictor and inclusion or omission of literature values. The explanatory variable “x” of this model is fresh or dry weight [mg] whereas the response variable “y” is the 48 h LD50 of dimethoate [μg a.i./bee]. Parameter “a” is the slope of the function and “b” its intercept with the y-axis. Model function can be alternatively expressed as *y* = 10^*b*^ · *x*^*a*^.
