# Supplementary material for: Interspecific sensitivity of bees towards dimethoate and implications for environmental risk assessment
Source: Sci Rep. 2016 Sep 30;6:34439. doi: 10.1038/srep34439 (PMC5043368; doi:10.1038/srep34439)
Supplement: Supplementary Information [file srep34439-s1.pdf]

Supplementary Information

Interspecific sensitivity of bees towards dimethoate and implications for risk assessment

Philipp Uhl[1], Lea A. Franke[1], Christina Rehberg[1], Claudia Wollmann[1], Peter Stahlschmidt[1], Lukas Jeker[2,3], Carsten A. Brühl[1]

[1]Institute for Environmental Sciences, University of Koblenz-Landau, Fortstrasse 7, 76829 Landau, Germany

[2]Dr. Knoell Consult Schweiz GmbH, Riehenstrasse 43, 4058 Basel, Switzerland

[3]Eidgenössisches Departement für Wirtschaft, Bildung und Forschung WBF, Agroscope, Zentrum für Bienenforschung, Schwarzenburgstrasse 161, 3003 Bern, Switzerland (current affiliation)

email: uhl@uni-landau.de

Collection and identification of wild bees

Wild bees bee were caught at feeding grounds or nesting sites. Three species were found in sufficient numbers: *L. malachurum*, *A. flavipes* and *C. hederae* (Fig. S1). All of them are nesting in aggregations and are therefore quite abundant at their nesting sites (Bischoff et al. 2005, Westrich 1990). Bees were either collected from flowers with small plastic cups or caught with a hand net between morning and midday. Afterwards, bees were directly anaesthetised by cooling and transferred to group cages where they were fed 50% sugar solution ad libitum. *L. malachurum* collected in a vineyard (8°5'19"E / 49°7'47"N), *A. flavipes* on a wildflower meadow (8°11'32"E / 49°8'57"N) and *C. hederae* at a small loess wall behind a vineyard (8°9'53"E / 49°13'28"N). After the termination of the experiments the surviving wild bees were killed by freezing and species were accurately determined. Precise identification was only possible with dead bees under a binocular. Studied specimen proved to be of the desired species except for two individuals that were actually *L. pauxillum* and *L. calceatum* instead of *L. malachurum*.

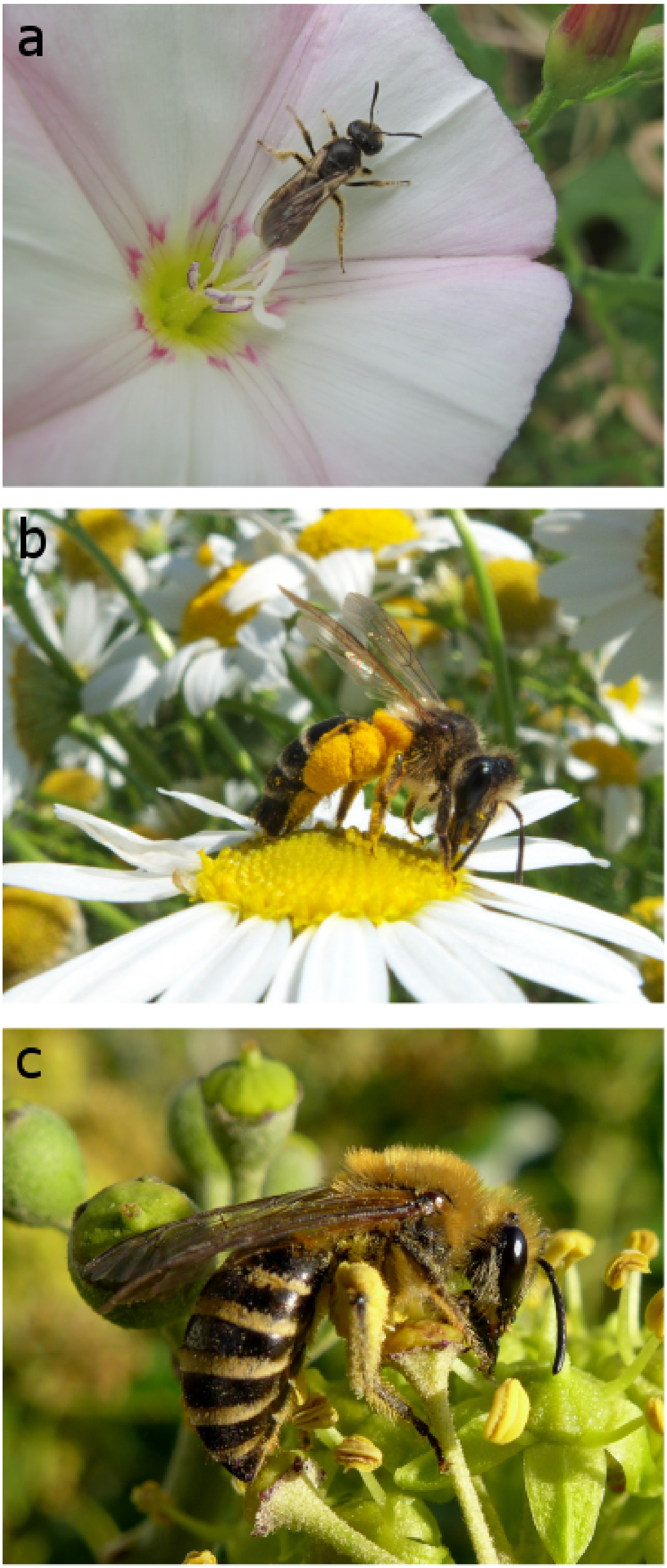

Figure S1: Captured bee species. *L. malachurum* ♀ in a field bindweed flower (a), *A. flavipes* ♀ on feverfew flower (b), *C. hederae* ♀ collecting pollen on ivy (c). Photo credit: Carsten A. Brühl.

Dose verification of treatment solutions

Samples of the highest and lowest concentrated treatment solutions were taken in all *B. terrestris* and *O. bicornis* ♀ test runs and stored in a freezer at -21 ℃ until shipping. They were shipped to Ivo Roessink (Alterra, Wageningen UR, 6700AA Wageningen, The Netherlands) for quantitative HPLC analysis of their dimethoate content (Table S1). Only test runs were the measured dimethoate concentration was within ± 5% of the nominal concentration were deemed valid.

Table S1: Results of analytical dose verification.

Species	Test run	Dimethoate concentration [µg/µL]		Deviation [%]
		nominal	measured	
B. terrestris	1	0.2500	0.2543	+1.74
		4.0000	4.1770	+4.43
O. bicornis ♀	1	0.6250	0.6249	-0.16
		10.0000	10.2706	+2.71
	2	0.6250	0.5964	-4.58
		10.0000	9.8831	-1.17
	3	0.6250	0.6223	-0.43
		10.0000	10.2286	+2.29

Further information

Preparation of Calibration Solutions

The standard stock solution with a concentration of 250 mg/L DIMETHOATE was prepared in methanol using reference material with a purity of 98.5% (Dr. Ehrenstorfer). Stock solution was stored in the freezer at temperature lower than -10 ℃.

External calibration standards with concentrations between 2.5 and 500 ng/ml were freshly prepared prior analysis by diluting the stock solution with acetonitrile/Milli-Q water (20v/80v) directly in GC vials, using a dilutor Hamilton 600. Duplo samples of about 3.0 mL were taken from the dosage solutions by mean of a glass pipette and transferred into a 4-mL brown vial, containing 1.0 mL acetonitrile. After homogenizing using a vortex one of the duplo's was stored in the refrigerator at 4℃ (range 2 to 8℃) and the other one, was diluted prior to analysis with acetonitrile/MilliQ-water (20v/80v) and analyzed directly (without extraction or concentration) by means of LC-MSMS. The dilution has been done also directly in GC vials, using a dilutor Hamilton 600. Injected samples were quantified by dimethoate peak area using the calibration curve constructed from calibration standards included in the same sample sequence. The concentrations of the samples never exceeded the highest standard of the calibration curve. The curve fit was linear and forced through origin (x-axis zero; y-axis zero).

LC-MS/MS-Conditions for dimethoate

Instrument

Autosampler: Agilent G1329A

Pump: Agilent G1312A (binary pump)

Detector: Agilent G63110A QQQ

Source: Agilent G1948 Electrospray

Column thermostat: Agilent G1316A

Separation

Eluent A: MilliQ-water (Advantage A10) + 0.1% formic acid

Eluent B: Acetonitril + 0.1% formic acid

Gradient:	Time	%B
	0.0	60
	2.0	60
	3.0	80
	6.0	80
	7.0	60
	8.0	60

Injection Volume: 50 µL

Flow Rate: 0.7 mL/min

Column: Agilent Zorbax Eclipse XDB C18 (4.6 mm x 150 mm, 5 micron)

Column temperature: 40℃

Detection

Ionization Mode: Positive

Heater Gas Temperature: 350 ℃

Spray Voltage: 3000 V

Nebulizer pressure: 50 psi

Nitrogen flow: 10 L/min

Scan Mode: Multiple reaction monitoring (MRM)

Compound	Precursor Ion	Product Ion	Fragmentation	Collision Energy
dimethoate	230	198.8	60	5
dimethoate	230	171	60	9
dimethoate	239	125	60	21

Retention time: about 2.75 min.

LOD in the injected samples: 0.03 ng/mL

LOQ in the injected samples: 0.09 ng/mL

Table S2: Weight of studied bee species.

Species	Fresh weight [mg]	95% CI [mg]	Dry weight [mg]	95% CI [mg]
L. malachurum	10.97	4.28 – 17.66	3.71	1.34 – 6.09
O. bicornis ♂	37.69	7.19 – 68.19	17.61	4.58 – 30.63
A. flavipes	47.26	19.35 – 75.17	21.61	14.44 – 28.77
O. bicornis ♀	93.57	62.72 – 124.42	30.36	23.30 – 37.42
C. hederae	105.52	66.36 – 144.68	43.43	29.00 – 57.86
B. terrestris	205.01	116.84 – 293.19	55.79	29.53 – 82.04

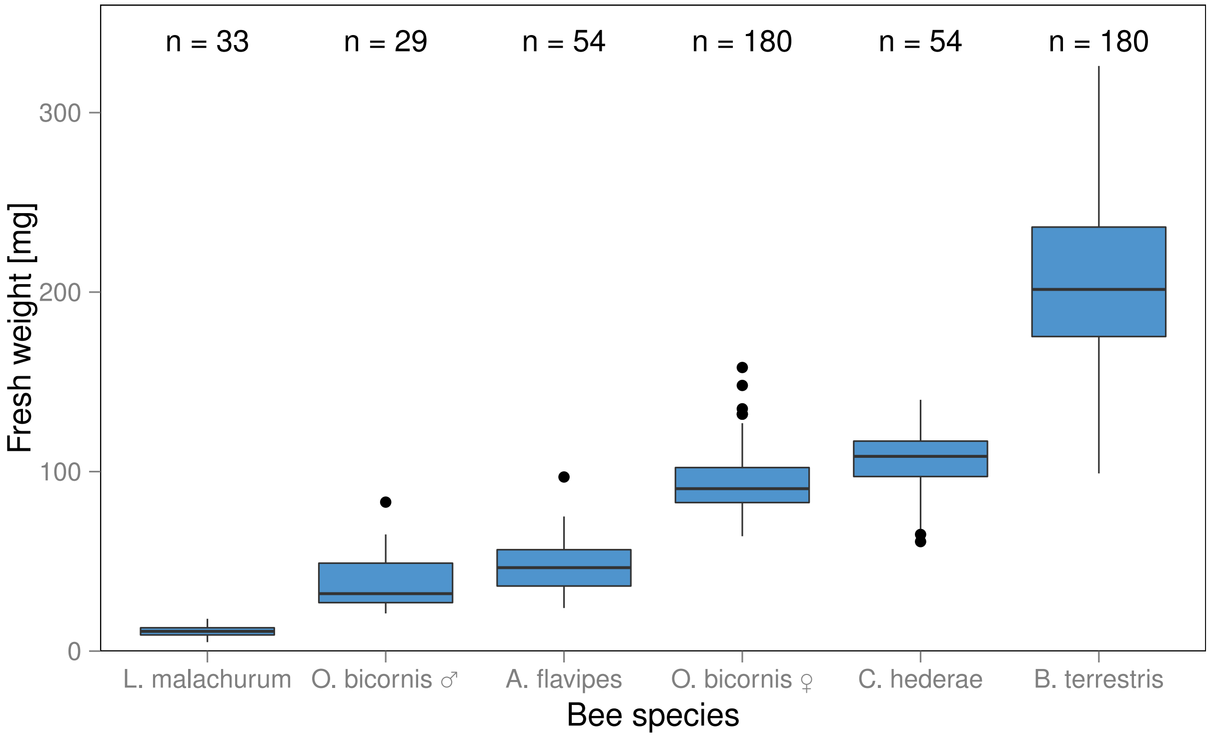

Figure S2: Boxplots of fresh weight for studied bee species.

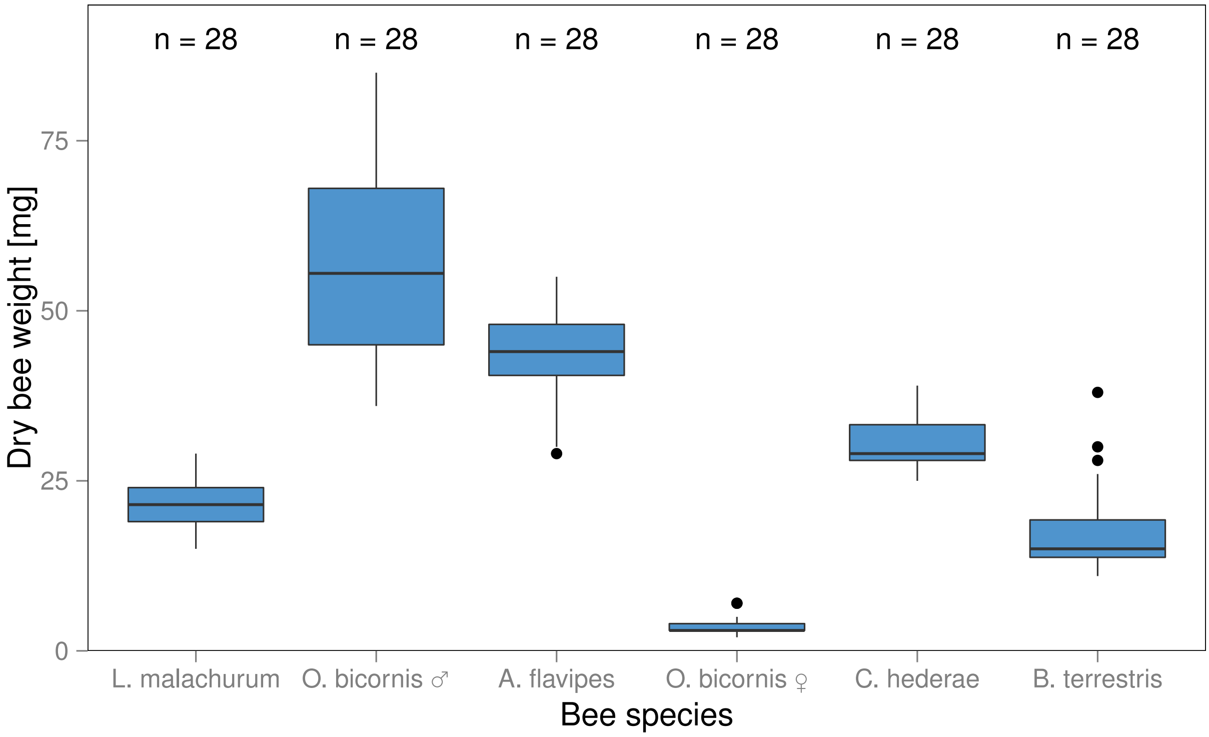

Figure S3: Boxplots of dry weight for studied bee species.

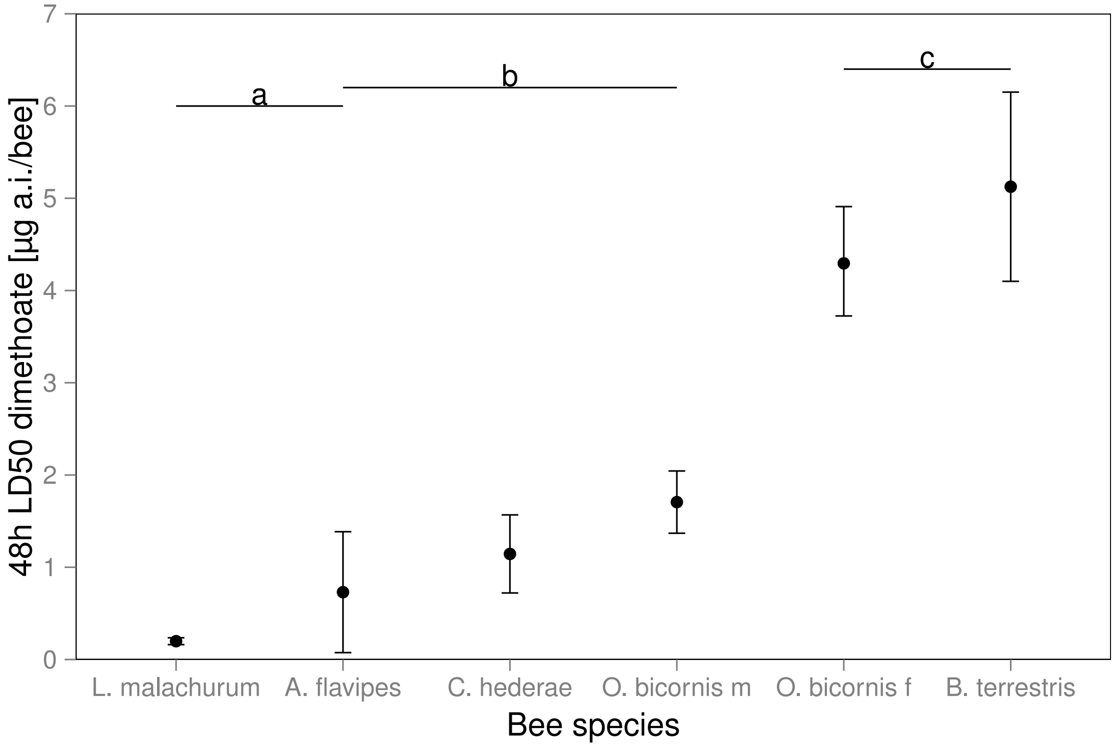

Figure S4: LD50 values for the studied bee species. Means and 95% CIs. Letters indicate statistical significance ($p < 0.05$).

Table S3: Comparison of 48 h dimethoate LD50 values of studied bee species

Compared species		Mean difference	95% CI	p
Species 1	Species 2	[µg a.i./bee]	[µg a.i./bee]	
A. flavipes	*L. malachurum*	0.53	-0.38 – 1.44	0.086
C. hederae	*L. malachurum*	0.95	0.36 – 1.53	<0.001
O. bicornis ♂	*L. malachurum*	1.51	1.04 – 1.98	<0.001
O. bicornis ♀	*L. malachurum*	4.10	3.21 – 4.99	<0.001
B. terrestris	*L. malachurum*	4.93	3.51 – 6.35	<0.001
C. hederae	*A. flavipes*	0.41	-0.67 – 1.49	1
O. bicornis ♂	*A. flavipes*	0.98	-0.04 – 2.00	0.075
O. bicornis ♀	*A. flavipes*	3.56	2.30 – 4.83	<0.001
B. terrestris	*A. flavipes*	4.40	2.71 – 6.08	<0.001
O. bicornis ♂	*C. hederae*	0.56	-0.19 – 1.31	0.413
O. bicornis ♀	*C. hederae*	3.15	2.09 – 4.21	<0.001
B. terrestris	*C. hederae*	3.98	2.44 – 5.52	<0.001
O. bicornis ♀	*O. bicornis* ♂	2.59	1.59 – 3.59	<0.001
B. terrestris	*O. bicornis* ♂	3.42	1.92 – 4.91	<0.001
B. terrestris	*O. bicornis* ♀	0.83	-0.84 – 2.51	1

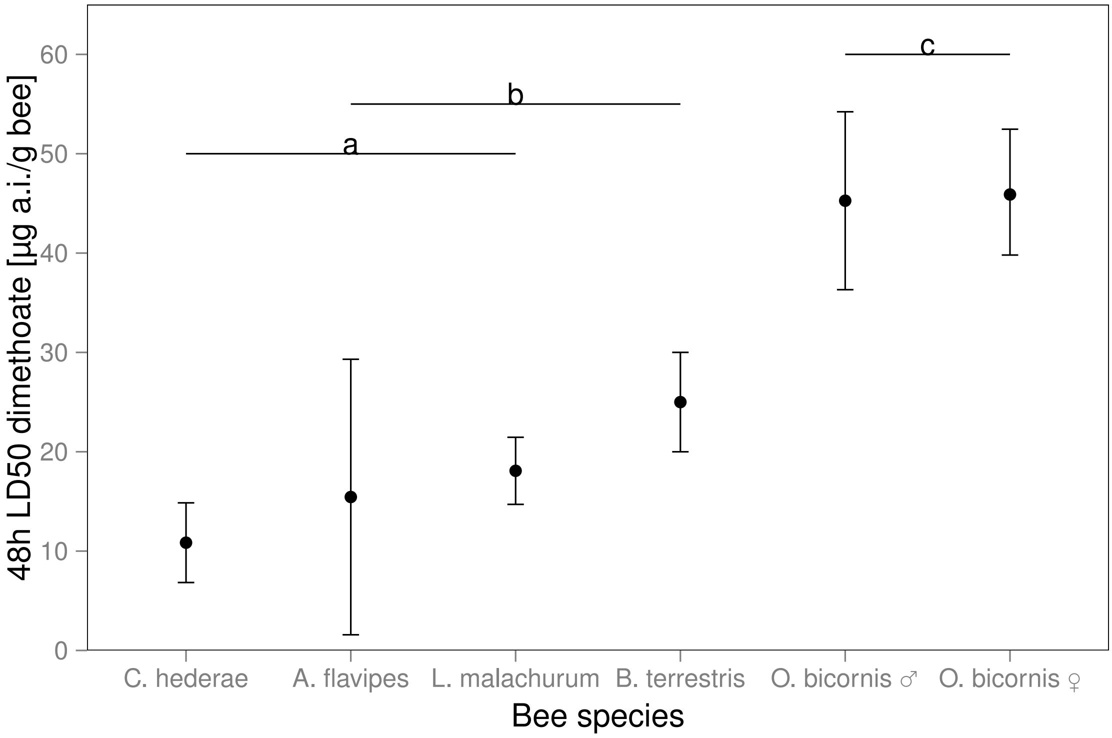

Figure S5: Fresh weight-normalised LD50 values for the studied bee species. Means and 95% CIs. Letters indicate statistical significance ($p < 0.05$)

Table S4: Comparison of fresh weight-normalised 48 h dimethoate LD50 values of studied bee species

Compared species		Mean difference	95% CI	p
Species 1	Species 2	[µg a.i./g bee]	[µg a.i./g bee]	
A. flavipes	*L. malachurum*	-2.64	-24.01 – 18.74	1
C. hederae	*L. malachurum*	-7.24	-15.09 – 0.62	0.103
O. bicornis ♂	*L. malachurum*	27.19	12.86 – 41.52	<0.001
O. bicornis ♀	*L. malachurum*	27.81	17.06 – 38.56	<0.001
B. terrestris	*L. malachurum*	6.92	-2.12 – 15.96	0.367
C. hederae	*A. flavipes*	-4.60	-26.22 – 17.02	8.152
O. bicornis ♂	*A. flavipes*	29.82	5.10 – 54.55	0.006
O. bicornis ♀	*A. flavipes*	30.45	7.62 – 53.28	0.002
B. terrestris	*A. flavipes*	9.56	-12.52 – 31.64	1
O. bicornis ♂	*C. hederae*	34.42	19.73 – 49.12	<0.001
O. bicornis ♀	*C. hederae*	35.05	23.82 – 46.28	<0.001
B. terrestris	*C. hederae*	14.16	4.55 – 23.76	<0.001
O. bicornis ♀	*O. bicornis* ♂	0.63	-15.80 – 17.05	1
B. terrestris	*O. bicornis* ♂	-20.27	-35.63 – -4.91	0.002
B. terrestris	*O. bicornis* ♀	-20.89	-32.98 – -8.81	<0.001

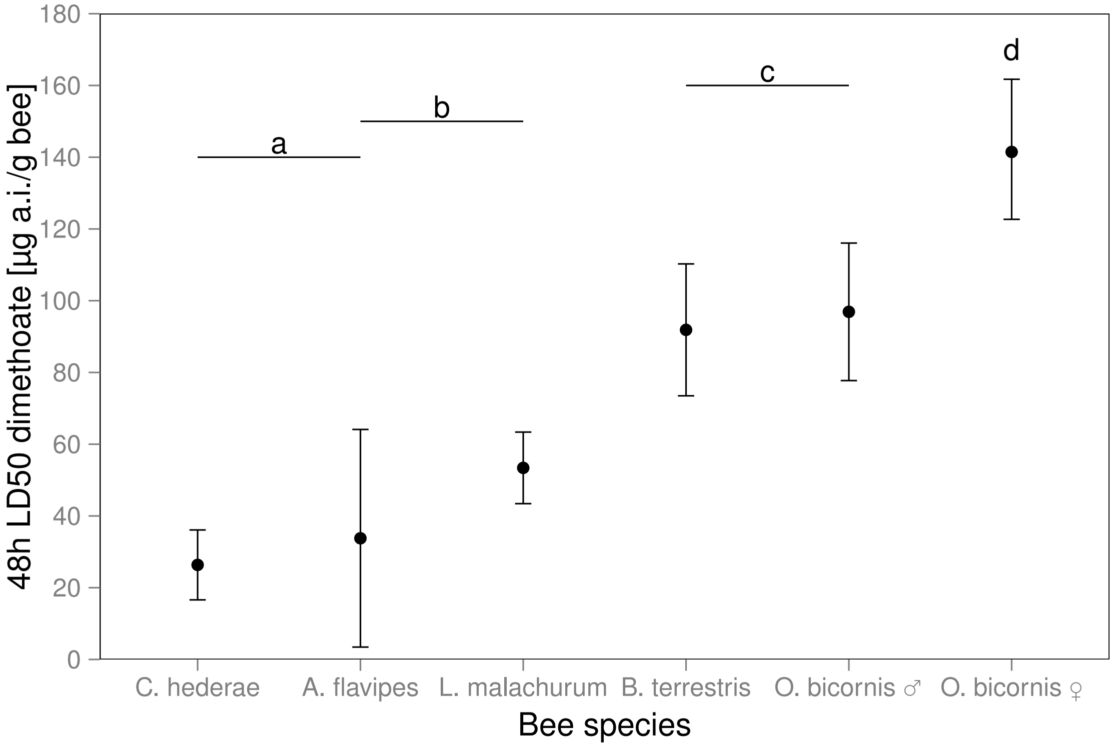

Figure S6: Dry weight-normalised LD50 values for the studied bee species. Means and 95% CIs. Letters indicate statistical significance ($p < 0.05$)

Table S5: Comparison of dry weight-normalised 48 h dimethoate LD50 values of studied bee species

Compared species		Mean difference	95% CI	p
Species 1	Species 2	[µg a.i./g bee]	[µg a.i./g bee]	
A. flavipes	*L. malachurum*	-19.62	-67.44 – 28.20	1
C. hederae	*L. malachurum*	-27.05	-47.93 – -6.17	0.002
O. bicornis ♂	*L. malachurum*	43.50	11.15 – 75.86	0.001
O. bicornis ♀	*L. malachurum*	88.07	55.23 – 120.90	<0.001
B. terrestris	*L. malachurum*	38.48	7.15 – 69.80	0.005
C. hederae	*A. flavipes*	-7.43	-55.14 – 40.28	1
O. bicornis ♂	*A. flavipes*	63.12	9.39 – 116.86	0.009
O. bicornis ♀	*A. flavipes*	107.69	53.66 – 161.71	<0.001
B. terrestris	*A. flavipes*	58.10	4.98 – 111.21	0.021
O. bicornis ♂	*C. hederae*	70.55	38.35 – 102.75	<0.001
O. bicornis ♀	*C. hederae*	115.11	82.44 – 147.79	<0.001
B. terrestris	*C. hederae*	65.52	34.36 – 96.68	<0.001
O. bicornis ♀	*O. bicornis* ♂	44.56	3.59 – 85.54	0.022
B. terrestris	*O. bicornis* ♂	-5.03	-44.80 – 34.75	1
B. terrestris	*O. bicornis* ♀	-49.59	-89.75 – -9.43	0.005

Table S6: Dimethoate SSD model information

Model type	Model parameters			
	Scale	SE	Location	SE
log-logistic	0.3514	0.0951	-0.0787	0.2090

Table S7: Contact dimethoate 48h LD50 values for bee species from literature

Species	LD50 [µg a.i./bee]	geometric mean LD50 [µg a.i./bee]	Source
A. mellifera	0.16		Ladurner et al. (2005)
A. mellifera	0.31	0.18	Biddinger et al. (2013)
A. mellifera	0.12		Stevenson (1968)
O. cornifrons	0.09	0.09	Biddinger et al. (2013)
O. lignaria	1.21	1.21	Ladurner et al. (2005)

References

Biddinger, D. J., Robertson, J. L., Mullin, C., Frazier, J., Ashcraft, S. A., Rajotte, E. G., Joshi, N. K. & Vaughn, M. (2013). Comparative toxicities and synergism of apple orchard pesticides to Apis mellifera (L.) and Osmia cornifrons (Radoszkowski). *PloS one*, 8(9):e72587.

Bischoff, I., Eckelt, E. & Kuhlmann, M. (2005). On the biology of the ivy-bee Colletes hederae Schmidt & Westrich, 1993 (Hymenoptera, Apidae). *BONNER ZOOLOGISCHE BEITRAGE*, 53(1/2):27.

Ladurner, E., Bosch, J., Kemp, W. P. & Maini, S. (2005). Assessing delayed and acute toxicity of five formulated fungicides to Osmia lignaria Say and Apis mellifera. *Apidologie*, 36(3):449.

Stevenson, J. H. (1968). Laboratory studies on the acute contact and oral toxicities of insecticides to honeybees. *Annals of Applied Biology*, 61(3):467–472.

Westrich, P. (1990). *Die Wildbienen Baden-Württembergs: [im Rahmen des Artenschutzprogrammes Baden Württemberg]*. Ulmer, Stuttgart, 2., verb. aufl. edition.
